# A Live Video Mind-Body Treatment to Prevent Persistent Symptoms Following Mild Traumatic Brain Injury: Protocol for a Mixed Methods Study

**DOI:** 10.2196/25746

**Published:** 2021-01-14

**Authors:** Jonathan Greenberg, Tanya Singh, Grant L Iverson, Noah D Silverberg, Eric A Macklin, Robert A Parker, Joseph T Giacino, Gloria Y Yeh, Ana-Maria Vranceanu

**Affiliations:** 1 Integrated Brain Health Clinical and Research Program Massachusetts General Hospital Harvard Medical School Boston, MA United States; 2 Department of Physical Medicine and Rehabilitation Spaulding Rehabilitation Hospital and Spaulding Research Institute Harvard Medical School Charlestown, MA United States; 3 MassGeneral Hospital for Children Sports Concussion Program Boston, MA United States; 4 Home Base A Red Sox Foundation and Massachusetts General Hospital Program Charlestown, MA United States; 5 Department of Psychology University of British Columbia Vancouver, BC Canada; 6 Rehabilitation Research Program Vancouver Coastal Health Research Institute Vancouver, BC Canada; 7 Harvard Medical School Boston, MA United States; 8 Biostatistics Center Massachusetts General Hospital Boston, MA United States; 9 Spaulding Rehabilitation Hospital Charlestown, MA United States; 10 Department of Psychiatry Massachusetts General Hospital Boston, MA United States; 11 Department of Medicine Beth Israel Deaconess Medical Center Boston, MA United States

**Keywords:** mild traumatic brain injury, anxiety, mixed methods, intervention development

## Abstract

**Background:**

Every year, approximately 42 million people sustain a mild traumatic brain injury (mTBI, also known as concussion), with particularly high rates among college-aged individuals. A substantial proportion of these people (44%-64%) develop persistent symptoms that are challenging to treat, costly, and associated with significant disability. Anxiety has emerged as a risk factor for progression from acute to persistent mTBI symptoms.

**Objective:**

This study aims to develop, adapt, and establish the feasibility of the *Toolkit for Optimal Recovery after Concussions* (TOR-C), an innovative mind-body program aimed at preventing persistent symptoms among young adults with mTBI and comorbid anxiety. Here, we describe the proposed study design, methodology, measurement, and treatment manuals.

**Methods:**

In phase 1, we will conduct individual, live video qualitative interviews (up to n=20) with college-aged individuals with mTBI and comorbid anxiety to inform adaptation of the intervention and study procedures. In phase 2, an open pilot of the live video TOR-C (n=5) with exit interviews will be conducted to explore the initial feasibility, acceptability, and credibility of the program and to refine the study procedures. Phase 3 will involve conducting a feasibility randomized controlled trial (N=50) of the TOR-C versus a health education control (Health Enhancement for Concussions; HE-C), both delivered via live video, to establish feasibility of recruitment procedures (screening, eligibility, and enrollment) and data collection; feasibility, credibility, and acceptability of the live video TOR-C and HE-C (adherence, retention, fidelity, and satisfaction) following prespecified benchmarks; and a signal of improvement in outcomes.

**Results:**

Phase 1 of the study has been approved by the Massachusetts General Hospital Institutional Review Board. Study completion is anticipated by early 2025.

**Conclusions:**

We will develop and test the first mind-body intervention focused on prevention of persistent symptoms following mTBI in young adults with comorbid anxiety problems. This will allow us to establish feasibility markers in postconcussive symptoms, anxiety, disability, and fear avoidance to inform a future efficacy trial of the TOR-C versus HE-C.

**International Registered Report Identifier (IRRID):**

PRR1-10.2196/25746

## Introduction

### Background

Every year, approximately 42 million people worldwide sustain a mild traumatic brain injury (mTBI), also known as concussion [1], with particularly high rates among college-aged individuals. Nearly all patients report physical (eg, headache), emotional (eg, irritability), behavioral (eg, insomnia), or cognitive (eg, difficulty concentrating) symptoms in the week following an mTBI [2]. Although many patients make a full recovery, a substantial proportion of patients experience persistent symptoms that can become intractable over time. Indeed, at 3 months and 1 year postinjury, up to 64% [3] and 44% [4] of patients with mTBI, respectively, continue to report 3 or more persistent symptoms [5]. Spontaneous symptom improvement is unlikely beyond this period [6]. It is therefore critical to provide early interventions to prevent symptom persistence.

Unlike moderate-to-severe traumatic brain injury (TBI), the prognosis of mTBI is not well correlated with injury severity or clinical findings [7]. Rather, symptom persistence after mTBI reflects a complex mind-body interaction, with anxiety playing a prominent role [7,8]. Anxiety may contribute to symptom persistence after mTBI by mimicking or amplifying symptoms, increasing hypervigilance and misattributions, and motivating activity avoidance [9]. These mechanisms are consistent with the fear avoidance theoretical model, which explains the transition from acute to chronic pain [10], and they provide a useful conceptualization for how anxiety causes or amplifies mTBI symptoms and leads to symptom persistence. We have previously shown that both catastrophizing and activity avoidance mediate the relationship between anxiety and postconcussion symptoms in patients with mTBI [11], supporting the applicability of the fear avoidance model to patients with mTBI.

For the substantial proportion of patients with mTBI and anxiety, current approaches are inadequate to prevent symptom persistence and disability. Accumulating evidence suggests that re-engagement in activities of daily living is healthy and promotes recovery, whereas rest and avoidance induces or maintains nonspecific symptoms and perpetuates activity avoidance [12,13]. For patients with anxiety, following recommendations for re-engagement is challenging because of maladaptive beliefs that rest is beneficial and activity is dangerous [14,15] as well as physiological manifestations of anxiety that mimic postconcussion symptoms [16]. As such, patients with anxiety and mTBI are at risk for decreased functioning across occupational, social, and recreational contexts and for experiencing persistent symptoms [17]. To date, there are no evidence-based psychosocial interventions for patients with recent mTBI (acute and subacute periods, up to 3 months postinjury [18]) and anxiety, which are focused on breaking the cycle of avoidance and preventing symptom persistence. Thus, it is critical to develop a prevention intervention that is feasible, accepted, and efficacious.

Mind-body interventions effectively treat both individual symptoms common to mTBI (eg, headache [19], insomnia [20], and fatigue [19]) and anxiety [21]. Furthermore, they do not carry stigma that is often associated with traditional mental health referrals [22] and are well tolerated and popular among patients with neurological conditions [23]. Mind-body interventions can also be effectively delivered via live videos [24,25]. Live video represents a promising avenue for delivering preventative care for individuals with acute mTBI and anxiety, who face many barriers to in-person visits, such as symptom burden, time and cost associated with travel, decreased flexibility in scheduling, and lower access to trained providers relative to live video delivery [26]. Importantly, live video delivery enables safe participation while maintaining social distancing, in line with guidelines issued by the Centers for Disease Control and Prevention [27] and the World Health Organization [28] following the COVID-19 pandemic.

College-aged adults with mTBI and comorbid anxiety are in high need of a live video mind-body intervention for several reasons. First, mTBIs are particularly common among this population [29-31]. Second, this age group has the highest rates of anxiety symptoms (approximately 40%), even in the absence of an injury [32]. Finally, college-aged individuals prefer live video conferencing over in-person interventions [33].

### Objectives

Our team has developed a brief live video mind-body treatment, the *Toolkit for Optimal Recovery after Injury* (*TOR*) [34], to prevent chronic pain in people with orthopedic injuries who have high pain-related anxiety or catastrophic thinking about pain. In this study, we propose to adapt this program for the unique needs of college-aged individuals with recent mTBI (ie, in the acute and subacute stages of recovery [18]) and anxiety (*Toolkit for Optimal Recovery after Concussion; TOR-C*), and iteratively optimize it using mixed methods. Our study has 3 phases: (1) qualitative live video interviews with college-aged individuals with recent mTBIs and anxiety (n=20) to identify their treatment needs and preferences and develop the live video TOR-C and study procedures; (2) an open pilot study (n=5) with exit interviews and pretreatment and posttreatment assessments to explore the initial feasibility, acceptability, and credibility of the live video TOR-C and study procedures; and (3) a pilot feasibility randomized controlled trial (RCT) of the TOR-C versus Health Enhancement for Concussions (HE-C), both delivered via live video (N=50), to establish the feasibility of recruitment procedures (screening, eligibility, and enrollment) and data collection as well as the feasibility, credibility, and acceptability of the live video TOR-C and education control condition (adherence, retention, fidelity, and satisfaction), following prespecified benchmarks. We hypothesize that the final version of the TOR-C will be feasible, accepted by patients, and associated with within-group improvements in postconcussive symptoms, anxiety, depression, disability, mindfulness, behavioral responses to illness, pain catastrophizing, and fear avoidance. This paper describes the study protocol.

## Methods

### Study Design

Our study design and methodology are informed by the Obesity Related Behavioral Intervention Trials (ORBIT) [35] and National Center for Complementary and Integrative Health (NCCIH) [36] models of intervention development, which emphasize the importance of iteratively optimizing interventions to establish feasibility markers before efficacy testing. Our primary outcomes will be feasibility, credibility, and acceptability markers to support a future efficacy RCT. Secondary outcomes are postconcussive symptoms, anxiety, disability, mindfulness, pain catastrophizing, behavioral responses to illness, and fear avoidance. See Multimedia Appendix 1 for peer review of this research proposal by the National Institutes of Health.

### Inclusion and Exclusion Criteria

We plan to include participants aged 18 to 24 years who have been diagnosed as having uncomplicated mTBI (ie, without intracranial abnormality) [37] 3 to 6 weeks earlier, score >8 on the Generalized Anxiety Disorder 7-Item Scale (GAD-7 scale; indicating at least mild-to-moderately elevated anxiety) [38], are fluent in English, and can participate in a live video interview (phase 1) or intervention (phase 2 and 3). Exclusion criteria include participation in mind-body or cognitive-behavioral therapy in the past 3 months, practice of mindfulness techniques >45 minutes per week on average in the past 3 months, current or previous history of complicated mTBI or moderate or severe TBI, change in psychotropic medications in the past 3 months, psychosis, bipolar disorder, active substance abuse or dependence, or pregnancy. As this is a feasibility study, we expect that these criteria may change over the course of the 3 phases to maximize feasibility markers.

### Recruitment and Sampling

We plan to use the same recruitment procedure and eligibility criteria for all phases of the study. Some modifications may occur based on qualitative feedback from participants and lessons learned. Patients will be enrolled primarily from the sports concussion clinic at Massachusetts General Hospital. Medical staff at the concussion clinic will refer patients with uncomplicated mTBI, that is, those who show no structural abnormality [37,39]. Neuroimaging is not a requirement for study entry, and clinical neuroimaging is relatively uncommon for patients attending the concussion clinic. In each phase, the medical team will confirm diagnoses and clear patients for participation in the study. A research assistant will screen potential participants and obtain informed consent. During the restrictions of COVID-19, all recruitment procedures will be performed remotely (eg, screening conducted by telephone, informed consent forms signed and sent electronically).

### Procedure

**Figure 1:**
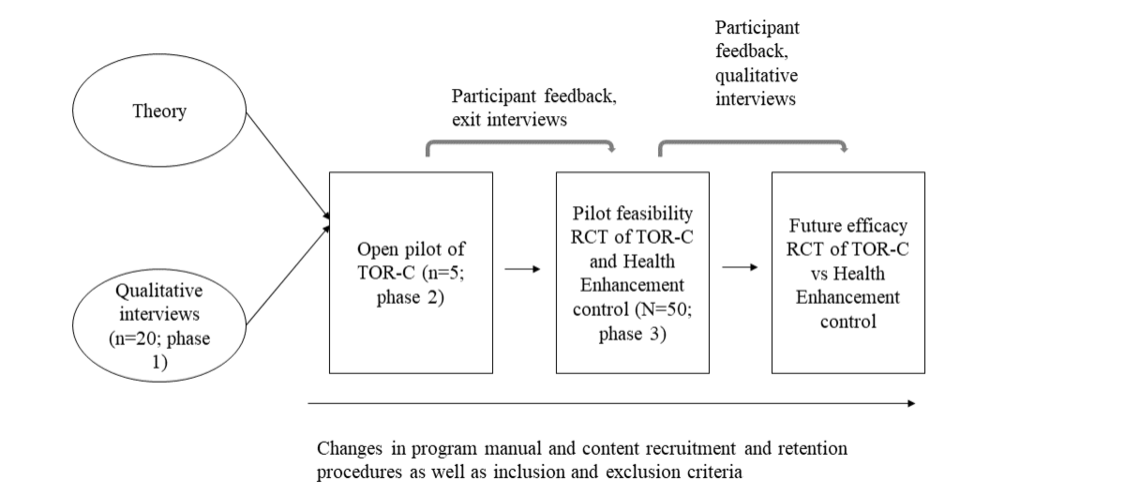
Phases of development of the Toolkit for Optimal Recovery after Concussion. RCT: randomized controlled trial; TOR-C: Toolkit for Optimal Recovery after Concussion.

Figure 1 depicts the iterative development and testing of TOR-C.

#### Phase 1: Development Phase

We will first develop a semistructured qualitative interview script to assess the specific treatment needs and preferences of young adults with mTBI. We will practice and pilot the semistructured interview over a secure video platform with 2 to 3 patients. We will subsequently administer the refined semistructured qualitative interview to 20 individuals with mTBIs to gather feedback on the intervention components and gauge treatment needs, expectations, and barriers. Specifically, the interview domains will include the following: (1) case-based scenarios of perceived effects of the injury and anxiety on function; (2) areas of need for skills training; (3) impact of the injury on activity, sports, school, work, and relationships; (4) best strategies for recruitment and retention for patients who have sustained concussions; (5) difficult situations or challenges experienced by college-aged individuals recovering from concussions; (6) barriers to participation and adherence, including light sensitivity and comfort using screens, and strategies to address this (eg, night setting, education); and (7) specific topics they would like to learn about. Participants will be provided information about the proposed content of the adapted program (TOR-C; Table 1) to obtain their feedback on each intervention component. Skills will be modeled to ensure an accurate understanding.

Eligible patients who agree to participate and provide written informed consent will be assisted in downloading and installing the video software (Zoom). All qualitative interviews will be audio-recorded, transcribed, organized, and analyzed using the NVivo 12 qualitative statistical package (QSR International; see the *Data Analyses* section). We will use the qualitative data obtained in phase 1 to develop the TOR-C and treatment manual.

**Table 1:** Proposed adaptations to the Toolkit for Optimal Recovery after Concussion intervention.

Original Toolkit (TOR)^a^		Adapted content for concussion (TOR-C)^b^
**Session 1**		
	Discuss rationale for targeting chronic pain; correct misconceptions about pain	Discuss rationale for targeting symptoms of concussion; correct misconceptions about recovery trajectory
	Learn physical, emotional, and cognitive factors that can speed or slow recovery after orthopedic injury; disability spiral for chronic pain	Learn physical (eg, sympathetic nervous system), emotional, and cognitive (negative automatic thoughts) factors that are conducive to symptom persistence after concussion. Discuss the overlap in symptom presentation between anxiety and concussion. Discuss the specific role of anxiety in persistence of symptoms after acute concussion (concussion specific disability spiral) and how skills can help break this spiral. Introduce avoidance and catastrophizing as key modifiable variables in the disability spiral. Discuss how TOR-C skills directly address these to facilitate recovery. Compare and contrast the disability spiral with the recovery spiral, with catastrophizing and avoidance as key
	Demonstrate relaxation strategies (diaphragmatic breathing and body scan) and their relevance to chronic pain	Provide education about the parasympathetic nervous system and relaxation; demonstrate relaxation strategies (diaphragmatic breathing, body scan) and their relevance to symptoms of concussion
	Set homework: practice relaxation strategies using recordings (20 min)	Set homework: practice relaxation strategies using recordings (20 min)
**Session 2**		
	Practice diaphragmatic breathing	Practice diaphragmatic breathing
	Review homework; barriers to practice	Review homework; barriers to practice
	Discuss bio-psychosocial model and mind-body links as they relate to pain	Discuss bio-psychosocial model and mind-body links as they relate to concussions and anxiety
	Identify thoughts-feelings-behaviors-sensations related to pain; mindfulness skills for habituating to pain sensations	Identify thoughts-feelings-behaviors-sensations related to concussion and anxiety symptoms; mindfulness skills for observing thoughts-feelings-behaviors nonjudgmentally to habituate to symptoms
	Identify negative or unhelpful thoughts about chronic pain; learn decision tree for negative or unhelpful thoughts regarding chronic pain	Identify negative or unhelpful thoughts about concussion and anxiety symptoms; general negative thoughts around catastrophizing and avoidance; learn decision tree for coping with negative or unhelpful thoughts regarding symptoms
	Provide reframing strategies; assist patients in reframing negative or unhelpful thoughts about chronic pain symptoms	Provide reframing strategies; assist patients in reframing negative and unhelpful thoughts about concussion symptoms
	Set homework: practice relaxation and mindfulness daily (new recording), complete decision tree and reframing exercises	Set homework: practice relaxation and mindfulness daily (new recording), complete decision tree and reframing exercises
**Session 3**		
	Practice diaphragmatic breathing	Practice diaphragmatic breathing
	Review previous material and homework; problem-solve barriers to practice	Review previous material and homework; problem-solve barriers to practice
	Assist patients in identifying a problem related to chronic pain; learn and apply problem-solving skills	Assist patients in identifying a problem related to concussion symptoms; learn problem-solving skills
	Learn acceptance strategies for chronic pain; assist patients in identifying when to use reframing versus problem solving versus acceptance	Learn acceptance strategies for concussion; assist patients in identifying when to use reframing versus problem solving versus acceptance
	Provide rationale for return to activity via activity pacing; assist patients in setting activity goals; assist patients in applying acceptance, reframing, or problem-solving skills to achieve pacing goals	Provide rationale for return to activity via activity pacing; assist patients in setting activity goals; assist patients in applying acceptance, reframing, or problem-solving skills to achieve activity pacing goals
	Set homework: practice relaxation strategies and mindfulness daily, complete decision tree exercise with options for problem solving, acceptance, and reframing; follow activity pacing protocol	Set homework: practice relaxation strategies and mindfulness daily, complete decision tree exercise with options for problem solving, acceptance, and reframing; follow activity pacing protocol
**Session 4**		
	Practice diaphragmatic breathing	Practice diaphragmatic breathing
	Review previous material and homework; problem-solve barriers to practice	Review previous material and homework; problem-solve barriers to practice
	Review all TOR skills; and identify which skills are being used and helpful, how helpful they are, and how they can be implemented in the future to help cope with chronic pain.	Review all TOR-C skills and identify which skills are being used, how helpful they are, and how they can be implemented in the future to help cope with concussion symptoms
	Interactive quiz to identify improvements, useful skills, and a plan for continued coping; continue practicing relaxation skills and mindfulness daily for 20 minutes using recordings	Interactive quiz to identify improvements patient has made, skills that are being used, skills the patient would like to continue to work on and a plan for continued coping; continue practicing relaxation skills and mindfulness daily for 20 minutes using recordings

^a^TOR: Toolkit for Optimal Recovery.

^b^TOR-C: Toolkit for Optimal Recovery after Concussion.

#### Phase 2: Open Pilot of TOR-C and Exit Interviews

We will enroll and treat up to 5 patients and conduct live video qualitative exit interviews at the end of the treatment to gather detailed feedback on the intervention components, applicability of the study measures, general protocol issues, and perceptions about live video intervention delivery. Participants will complete baseline, postintervention, and 3-month postintervention follow-up questionnaires as well as weekly home practice. We will thus be able to explore the feasibility and acceptability of the TOR-C, study measures and procedures, adherence to sessions and home practice, and acceptability of the live video in this population. Each session of the TOR-C will last 45 minutes. Exit interviews will last 30 minutes and be audio-recorded and transcribed. The interviews will follow procedures similar to those described in phase 1 (eg, delivery via a semistructured interview script, qualitative analysis using NVivo 12) and be used to further refine the study manual and procedures as well as finalize the TOR-C before the pilot RCT. The analysis plan is described in the *Data Analyses* section.

#### Phase 3: Feasibility RCT

After completing baseline questionnaires, eligible patients will be randomized via permuted blocks using a statistician-developed sequence to either the experimental (refined TOR-C) or control (HE-C) program in a 1:1 ratio. Participants will be blinded to intervention versus control groups. They will then be scheduled by a research assistant for their first video session. They will complete postintervention assessments after the 4-week intervention as well as at the 3-month follow-up. Both interventions will be delivered by the same mind-body clinician.

The treatment fidelity process for the RCT will follow the National Institutes of Health recommendations [40] and our previously successful clinical adherence protocol [41]. The clinicians will complete fidelity checklists after each session for both intervention and control groups and receive weekly supervision to ensure protocol adherence. To evaluate protocol fidelity for both the intervention and control, a random sample (10%) of the audio-recorded sessions will be coded by an independent coder.

### Live Video Delivery and Special Considerations for Patients With mTBI

The qualitative interview, open pilot, exit interviews, and feasibility RCT will all be delivered via a Health Insurance Portability and Accountability Act–approved video software, and all communication with participants and study staff will be performed remotely. On the basis of our previous work as well as prior research with this population and survey information from the concussion clinic at Massachusetts General Hospital [33], we anticipate that this live video format will be acceptable and well received by patients. However, we will specifically assess patients’ perceptions and preferences for live video in the qualitative interview (phase 1) and exit interviews following the open pilot (phase 2). Participants will be emailed a link to install the video software after enrollment. A research assistant will conduct test calls with participants and be available to assist them with any technical difficulties or challenges that they may experience during the interviews, open pilot, or feasibility RCT. We will use procedures established in prior virtual mind-body studies [41,42].

As some patients with mTBI experience light sensitivity [43], we will assess patients’ perceptions of live video delivery in phases 1 and 2. However, we do not expect this to be a barrier to live video delivery, as evidence from screen-based programs indicates that they are highly feasible and accepted by patients with mTBI, with no adverse light sensitivity issues reported [44-47]. We will consider adding educational information on light sensitivity to session 1 of the intervention, offering participants the option to use the *night setting* as a comfort measure.

### Program Structure and Modification

#### TOR-C Intervention

The original Toolkit [34] includes 4 manualized 45-min-long weekly sessions that address mind-body skills, including eliciting the relaxation response (eg, body scan, deep breathing, mindfulness), cognitive-behavioral strategies (eg, reframing), acceptance and commitment skills (eg, acceptance), and skills for returning to activity (eg, goal setting, activity pacing). Table 1 presents specific adaptations that we propose to make to each module for patients with mTBI and anxiety. These proposed adaptations will inform the qualitative interviews (phase 1), and additional refinements to the intervention will be made based on the results of our qualitative assessments.

#### HE-C (Control)

The HE-C will be adapted from the Health Enhancement Program [48], a manualized intervention that has been successfully used in prior live video studies [49,50]. It includes 4 modules that provide educational information on the relationship between anxiety and mTBI (session 1), return to activity (session 2), roles of nutrition and sleep (session 3), and health care self-management, including access to mental health services (session 4). This control condition will be dose-, attention-, and time-matched to the TOR-C in terms of session content and home practice. Specifically, participants in HE-C will be assigned daily readings [48] for the same amount of time as those in the intervention. Participants in the TOR-C will receive HE-C information as electronic handouts. Participants in both groups will have the option to opt in to receive text messages through EZ texting [51], a communication platform, which will remind them of dates and times for scheduled sessions to maximize attendance. Our team is using EZ texting successfully in projects with other medical populations [52,53].

### Assessments

In phases 2 and 3, patients will complete the following battery of reliable and valid questionnaires on the internet via Research Electronic Data Capture (REDCap), a secure web-based portal at baseline, postintervention (ie, after completing the 4-week active or control intervention), and 3-month follow-up.

#### Postconcussive Symptoms

This will be measured using the *Post-Concussion Symptom Scale* [54], a 22-item questionnaire measuring perceived presence and severity of concussion symptoms (eg, headache, nausea, balance problems, fatigue irritability, nervousness) on a 0-6 scale. Higher scores represent worse symptoms.

#### Anxiety and Depression

The GAD-7 [38] is a 7-item questionnaire measuring anxiety symptoms within the past 2 weeks on a scale of 0 to 3 and will be used to ensure participants meet the criteria for anxiety symptoms. The *Hospital Anxiety and Depression Scale* [55] is a 14-item questionnaire assessing anxiety and depression in the previous week on a scale of 0 to 3 and will be used to assess changes in anxiety symptoms following the intervention and at follow-up. Higher scores represent higher anxiety.

#### Disability

This will be measured using the World Health Organization Disability Assessment Schedule (WHODAS) 2.0 for mTBI [56,57], a 12-item questionnaire assessing functional difficulties in various life domains (eg, participation barriers, physical activity limitations, and self-care limitations) on a scale of 0 to 4. Higher scores represent lower physical function.

#### Fear Avoidance

This will be measured via the *Fear Avoidance Behavior after Traumatic Brain Injury Questionnaire* [58], a 16-item questionnaire assessing beliefs about how work and physical activities affect mTBI symptoms, and whether they should be avoided, on a scale of 0 to 3. Higher scores represent higher fear avoidance.

#### Pain Catastrophizing

This will be measured using the *Pain Catastrophizing Scale* [59], a 13-item questionnaire assessing one’s tendency to focus on pain-related thoughts and feel helpless and hopeless due to pain on a scale of 0 to 4. Higher scores indicate higher pain catastrophizing.

#### Mindfulness

This will be measured via the Cognitive and Affective Mindfulness Scale-Revised, a 12-item questionnaire assessing one’s ability to pay attention to the present moment in a nonjudgmental manner [60], on a scale of 1 to 4. Higher scores represent higher self-reported mindfulness.

#### Behavioral Response to Illness

This will be measured using the *limiting behavior* (7 items) subscale of the Behavioral Response to Illness Questionnaire [61], assessing the frequency in which participants are inactive as well as the *all or nothing behavior* (6 items) subscale, which captures one’s tendency to overexert themselves. Both subscales are rated on a scale of 0 to 4, with higher scores indicating more limiting and all or nothing behavior.

#### Treatment Satisfaction (Postintervention Only)

This will be assessed using the *Client Satisfaction Questionnaire* [62], a 3-item questionnaire assessing the degree to which the program met the participants’ needs and their satisfaction from it, on a scale of 1 to 4. Higher scores represent higher satisfaction.

#### Treatment Credibility (After Randomization Only)

This will be assessed using the *Credibility and Expectancy Questionnaire* [63]*,* a 6-item questionnaire that assesses how believable, convincing, and logical patients perceive the treatment to be and the degree to which they expect to improve. Some items are scored on a scale of 1 to 9, and others are scored on an 11-point 0%-100% scale. Higher scores represent higher credibility and expectancy.

#### Other Measures

We will additionally collect baseline data about other factors that may influence recovery and mTBI symptom persistence, such as smoking, alcohol consumption, time since injury, and posttraumatic headache. To minimize patient burden, these data will be assessed using 1 to 2 self-report questions (eg, “how many drinks do you consume a week on average?”) rather than full questionnaires. Additional information on medical history will be collected directly from patients’ electronic medical records. All these data, however, will not be included in our main analyses because of the current emphasis on feasibility. Rather, they will help us better characterize the sample and provide valuable information toward a future efficacy trial using the NCCIH UG3/UH3 mechanism. It is anticipated that patients will need 20 to 30 minutes to complete the battery of questionnaires. They will also receive a prompt at the end of each questionnaire, asking them to complete unanswered fields, in effort to reduce the possibility of missing data. A blinded research assistant who is not involved in recruitment procedures will check each questionnaire upon completion to ensure that patients do not randomly respond or acquiesce. To maintain confidentiality, patients will be identified using a specific ID. Only the unblinded research assistant and study clinicians will have access to the file connecting patients’ names and study IDs.

### Data Analyses

#### Phase 1 (Qualitative Interviews)

The individual qualitative interviews (phase 1) will be recorded and transcribed. They will then be analyzed using thematic content analyses following Miles and Huberman [64] in NVivo 12. We will use the framework method [65] to analyze our qualitative data and employ a primarily deductive approach [66], while also allowing flexibility to incorporate inductive themes derived directly from novel information collected during the interviews. We will assess the reliability (κ) of coding for themes and patterns in qualitative responses by 2 independent coders. The analyses will be conducted by trained clinicians and research assistants under the guidance of the research team’s senior psychologist. Discrepancies will be resolved through discussion until adequate reliability is obtained (κ>0.80).

#### Phases 2 (Open Pilot) and 3 (Feasibility RCT)

The primary analyses in this project will focus on establishing key quantitative feasibility benchmarks to inform future multisite RCTs. To this end, we will use frequency and proportions to assess the feasibility of recruitment and retention procedures within each group. We will additionally use proportions of patients with scores over the midpoint on the Client Satisfaction Questionnaire [62] and the Credibility and Expectancy Questionnaire to assess satisfaction and credibility, respectively. Feasibility benchmarks for these phases are detailed in Table 2.

**Table 2:** Feasibility benchmarks for phases 2 and 3.

Outcome	Acceptable	Excellent
Feasibility of recruitment	>70% of patients successfully contacted agree to participate	At least 80% of patients successfully contacted agree to participate
Credibility and expectancy	>70% of participants with score over scale midpoint	>75% of participants with score over scale midpoint
Client satisfaction score	>70% of participants with score over scale midpoint	>75% of participants with score over scale midpoint
Acceptability of treatment	>70% of participants attend 3 out of 4 sessions	>80% of participants attend 3 out of 4 sessions
Therapist adherence	>70% adherence (checklist and audio recordings)	100% adherence (checklists and audio recordings)
Adherence to homework	>70% of participants practice at least one skill on 3 days per week	>80% of participants practice at least one skill on 3 days per week
Feasibility of assessments	>70% of participants have no measures fully missing	>90% of participants have no measures fully missing
Adverse events	Minimal (see *Live Video Delivery and Special Considerations for Patients With mTBI* for light sensitivity issues)	None

Participants who drop out will be counted as not meeting applicable feasibility criteria. Benchmarks that need to be met before beginning the future fully powered RCT will include (1) >70% of participants successfully contacted agree to participate; (2) 70% of participants with a Credibility and Expectancy score and Client Satisfaction score over each scale’s midpoint; (3) >70% of participants participated in at least three of four sessions; (4) >70% therapist adherence (measured via checklists and independent rater agreement on audio recordings); (5) >70% of participants practice at least one skill on 3 days per week (6) >70% of participants have no questionnaires missing; and (7) minimal adverse events. If these criteria are not met, revisions will be necessary. Benchmarks will be reported separately for the TOR-C and HE-C. These benchmarks were used previously in federally funded studies by our research team [34,52,53] and are consistent with guidelines for intervention development [35,67].

For quantitative measures, we will use descriptive statistics to characterize the sample and paired sample two-tailed *t* tests to assess within-group changes between baseline and postintervention and between postintervention and the 3-month follow-up. Cohen *d*, including 95% CI, will be used to determine effect sizes using conventional standards (small effect sizes of 0.2 SD units, medium effect sizes of 0.5 SD units, and large effect sizes of 0.8 SD units) [68]. In line with recommendations for analyses in pilot studies [69,70], we will refrain from conducting between-group analyses of efficacy.

Analyses of the semistructured qualitative exit interviews in phase 2 will broadly follow the methods described in phase 1 above and will gather information on (1) perception of the skills taught in the program, (2) barriers and facilitators to completion of program and home practice, (3) perception of program structure and experience in the sessions, and (4) perception of the assessments.

#### Power Analysis

Power analysis is not appropriate for qualitative analyses. For the individual interviews, a sample size of n=20 is typically enough to achieve saturation of themes [71]. With a sample size of N=50 in phase 3 and conservatively assuming that the feasibility criteria in Table 2 are independent, the study will have 80% power to confirm feasibility of all criteria if the expected rate of each criterion is at least 83%. Of note, we achieved greater retention in some of our other studies, including the TOR study [34] as well as previous mind-body studies using live video [41]. Importantly, as mentioned above, this power analysis is not calculated to detect group differences or significant changes in outcome measures but rather to establish feasibility.

## Results

This study is funded by the NCCIH grant #K23AT01065301A1. Phase 1 was approved by the institutional review board of the Massachusetts General Hospital. Recruitment is due to start in December 2020 for phase 1 and in September 2021 and September 2022 for phases 2 and 3, respectively. Data collection for the feasibility RCT is anticipated to be completed by September 2024, and data analysis is anticipated to be completed by early 2025.

## Discussion

Anxiety is common among college-aged individuals and has emerged as one of the strongest modifiable risk factors for progression from acute to persistent mTBI symptoms [7,8]. Identifying individuals with acute mTBI and comorbid anxiety and enrolling them in a live video mind-body program may be an effective and efficient way to prevent costly and challenging-to-treat chronic symptoms following mTBI. In this paper, we describe the steps and study procedures for developing, adapting, and establishing the feasibility of the TOR-C, the first mind-body program aimed at preventing persistent mTBI symptoms among college-aged adults with mTBI and comorbid anxiety and delivered via live video. By applying a multimodal approach that combines mind-body skills (eg, deep breathing, mindfulness), cognitive-behavioral skills (eg, behavioral activation, reframing), and acceptance and commitment skills (eg, acceptance), the TOR-C may help break the negative cycle in which anxiety following mTBI leads to avoidance and thus perpetuates disability, symptom persistence, and further anxiety [9,10].

The results of this trial will provide important information toward a multisite RCT comparing the TOR-C with an HE-C control group. This is in line with the ORBIT [35] and NCCIH [36] intervention development models, emphasizing the importance of iteratively refining interventions and establishing feasibility benchmarks to ensure the scientific rigor of subsequent efficacy trials. This process is necessary to prevent common and negative consequences of leaping to efficacy testing before establishing feasibility, including inadequate fit of the intervention and/or procedures to the target population, lack of power to detect change, and inability to identify those who are likely to be most responsive to the intervention [72]. Moreover, processes in this study such as identifying treatment needs, preferences, perceptions, and barriers to treatment among these patients may further inform other treatments and interventions for this patient population.

In summary, this study will develop, adapt, and establish the feasibility of the TOR-C, the first mind-body intervention focused on *prevention* of persistent mTBI symptoms. It will be the first program specifically adapted and refined based on qualitative interviews to meet the unique needs, preferences, and challenges faced by this population, thus increasing its likelihood of efficacy. The results will inform a future multisite trial of TOR-C versus an HE-C control group and will potentially inform other interventions for this patient population. Future studies should also explore whether TOR-C is applicable to a wider range of patients with mTBI, including individuals across the life span.

## Appendix

Multimedia Appendix 1 Summary statement: National Institutes of Health peer review of funded grant.

## Abbreviations

GAD-7: Generalized Anxiety Disorder 7-item scale
HE-C: health enhancement for concussion
mTBI: mild traumatic brain injury
NCCIH: National Center for Complementary and Integrative Health
ORBIT: Obesity Related Behavioral Intervention Trials
RCT: randomized controlled trial
TBI: traumatic brain injury
TOR: Toolkit for Optimal Recovery After Injury
TOR-C: Toolkit for Optimal Recovery after Concussion
