# Supplementary material for: A Live Video Mind-Body Treatment to Prevent Persistent Symptoms Following Mild Traumatic Brain Injury: Protocol for a Mixed Methods Study
Source: JMIR Res Protoc. 2021 Jan 14;10(1):e25746. doi: 10.2196/25746 (PMC7843203; doi:10.2196/25746)
Supplement: Multimedia Appendix 1 [file resprot_v10i1e25746_app1.pdf]

SUMMARY STATEMENT
(Privileged Communication)

PROGRAM CONTACT:
Dr. Lanay Mudd
301-594-9346
muddlm@mail.nih.gov

Release Date: 08/13/2019
Revised Date:

Application Number: 1 K23 AT010653-01

Principal Investigator

GREENBERG, JONATHAN

Applicant Organization: MASSACHUSETTS GENERAL HOSPITAL

Review Group: ZAT1 JM (05)
National Center for Complementary and Integrative Health Special Emphasis Panel
NCCIH Training, Fellowship, and Career Development Review Panel (CT)

Meeting Date: 07/19/2019
Council: OCT 2019
Requested Start: 09/01/2019
RFA/PA: PA19-118
PCC: MUDDL

Project Title: Live video mind-body treatment to prevent persistent symptoms following mTBI

SRG Action: ++
Next Steps: Visit https://grants.nih.gov/grants/next_steps.htm
Human Subjects: 30-Human subjects involved - Certified, no SRG concerns
Animal Subjects: 10-No live vertebrate animals involved for competing appl.
Gender: 1A-Both genders, scientifically acceptable
Minority: 1A-Minorities and non-minorities, scientifically acceptable
Age: 7U-Only Adults, scientifically unacceptable

Project Year	Direct Costs Requested
1	146,372
2	146,372
3	146,372
4	146,372
5	146,372
TOTAL	731,860

++NOTE TO APPLICANT: Members of the Scientific Review Group (SRG) were asked to identify those applications with the highest scientific merit, generally the top half. Written comments, criterion scores, and preliminary impact scores were submitted by the assigned reviewers prior to the SRG meeting. At the meeting, the more meritorious applications were discussed and given final impact scores; by concurrence of the full SRG, the remaining applications, including this application, were not discussed or scored. The reviewers' comments (largely unedited by NIH staff) and criterion scores for this application are provided below. Because applications deemed by the SRG to have the highest scientific merit generally are considered for funding first, it is highly unlikely that an application with an ND recommendation will be funded. Each applicant should read the written critiques carefully and, if there are questions about the review or future options for the project, discuss them with the Program Contact listed above.
FOREIGN

1K23AT010653-01 Greenberg, Jonathan

DESCRIPTION (provided by applicant): This K23 proposal details a comprehensive 5-year training program that will support the candidate's transition toward an independent clinical research career focused on the development and rigorous testing of mind-body interventions with an emphasis on prevention of transition from acute to chronic symptoms following illness or injury. In this application the candidate proposes a significant and innovative proposal that is directly tied with his proposed training and career development goals. Background: College-age individuals have particularly high rates of concussions (also known as mTBIs) and anxiety. When these co-occur, there is an increased risk for transition to chronic mTBI symptoms, which are costly and challenging to treat. To date, there are no evidence-based interventions focused on preventing chronic mTBI. The Toolkit for Optimal Recovery (TOR) is a brief, live video mind-body program aimed at preventing chronic pain in patients with acute injuries, which is amenable for adaptations for the needs of college-age individuals with mTBI and anxiety. Specific aims and research design: The proposed study aims to 1) identify treatment needs and preferences among college-age individuals with acute mTBI and anxiety via live video qualitative interviews (N = 20) to inform adaptation of the TOR and study procedures; 2) explore, via an open pilot (N=5) with exit interviews and pre- and post- self-report assessments, initial feasibility, acceptability, and credibility of the live video TOR-Concussions (TOR-C) and study procedures, and to further refine the intervention, as needed; and 3) establish, via a pilot feasibility RCT of the TOR-C versus a matched dose, time and attention control (N = 60 enrolled), the feasibility, acceptability and credibility of both programs following pre- determined benchmarks. Findings will inform an efficacy trial through the UG3/UH3 or U01 mechanisms, as well as future studies aimed at generalizing this work to other patient populations. Training and mentoring: Study aims are supported by 3 main training goals aimed at developing expertise in: 1) qualitative methods; 2) technology-based interventions, and 3) mind-body intervention development and the conduct of rigorous clinical trials. These training goals are supported by 1) a team of expert mentors (Vranceanu & Yeh), collaborators (Iverson & Macklin), and contributors (Silverberg) that will oversee the progress of the project, 2) a rich institutional environment, and 3) targeted coursework, scientific meetings, seminars and planned publications. Relevance to the NCCIH mission. This K23 proposal is in line with NCCIH's funding priorities to develop feasible and acceptable mind-body interventions for difficult problems focused on prevention. Impact: Collectively, the experience gained during this award will serve as the foundation for the applicant's independent academic career in mind-body medicine, and will start a line of research focused on the development and testing of brief, feasible and scalable mind-body interventions with a focus on prevention of chronic illness.

PUBLIC HEALTH RELEVANCE: The proposed study will establish the feasibility, acceptability and credibility of a novel, live video mind-body program, "Toolkit for Optimal Recovery after Concussions; TOR-C", aimed at preventing persistent symptoms following concussion among college-age individuals with anxiety. This age group is particularly prone to suffer concussions (aka mTBI), and up to 40% have comorbid anxiety. As current treatments do not fully meet the needs of these patients, and miss the window of opportunity to prevent transition toward costly and challenging-to-treat mTBI with persistent symptoms, this proposal has the potential to dramatically improve outcomes in this population.

CRITIQUE 1

Candidate: 3
Career Development Plan/Career Goals /Plan to Provide Mentoring: 5
Research Plan: 5
Mentor(s), Co-Mentor(s), Consultant(s), Collaborator(s): 3
Environment Commitment to the Candidate: 3

Overall Impact:
This application is from a postdoctoral fellow at Massachusetts General Hospital (MGH) who is an experienced practitioner and researcher on mindfulness practices. The candidate would adapt an existing live video mindfulness intervention to a new population, patients with mild traumatic brain injury (mTBI) and collect pilot data before conducting a randomized trial with 60 college-age patients assigned to treatment or an active parallel control group. The candidate has three training goals: 1) to acquire training in qualitative methods, 2) develop expertise in technology-based interventions, and 3) develop expertise in mind-body intervention development and randomized clinical trials (RCTs). This candidate has a strong clinical background in mind-body practices and is a gifted clinical teacher and research supervisor. His background appears strong for launching an independent career. He recruited three mentors with complementary backgrounds and would have access to strong institutional research resources at MGH and Harvard University, supportive of mind-body practices and with access to a mTBI population. The enthusiasm for this application is dampened by several factors. The training goals are not ambitious in that they only slightly expand his existing expertise. There was too little attention paid to training in advanced statistics and in measurement issues. The research plan omits discussion of some critical variables (e.g. smoking, alcohol use, and headaches). MGH's and Harvard University's commitments to the candidate appeared weak, although a letter says he will be offered a three-year contract even if an award is not received.

1. Candidate:
Strengths
- The candidate has had considerable research productivity, with 14 publications since completing his PhD in clinical psychology in 2013.
- He has already designed and independently implemented several experimental studies on mindfulness, resulting in publications and praise from his mentors.
- The reference letters attested to his independence, leadership, and ability to problem-solve in recruitment and protocol problems with difficult populations. His letters praise his ability to train and manage research assistants, mentor laboratory personnel, and be an influential teacher.
- His enthusiasm for a research career in developing mindfulness interventions and understanding the mechanisms by which mindfulness achieves results is clearly based on his 15 years as a mediation practitioner and teacher.
- One mentor described his skills in statistical methods and computer programming as 'self-taught', demonstrating a commitment to a research career.

Weaknesses
- While the candidate has performed statistical analyses for prior publications and has taught courses on statistical inference and his training has been described as 'self-taught', he does not plan statistical coursework and training and no mentor will advise on statistics. The mentors did not address whether his statistical skills are adequate.
- The candidate's ability in teaching and using clinical approaches based on mindfulness practice was well-described. However, there was little discussion of the candidate's clinical expertise outside these practices and it appears his interests are quite narrow rather than broadly balanced.

2. Career Development Plan/Career Goals & Objectives:
Strengths
- The candidate's prior research experience has been very appropriate to his research interest and very strong in some areas. The candidate has recruited patients to clinical studies, has been a study clinician on studies using meditation-based approaches, has conducted research with healthy patients and those with affective conditions (e.g. depression and anxiety), and has learned neuroimaging techniques to understand the biological mechanisms associated with change in mind-body practices.

- The candidate’s goal to learn qualitative methods (i.e. interview studies) to inform the development of interventions is well-thought out and strong.
- The candidate’s plans to gain expertise in intervention development and the conduct of RCTs are appropriate and strong. He would conduct a pilot study with five individuals, adjust methods, and recruit and conduct a RCT with 60 individuals.
- There is good balance in training and research activities. The candidate would participate in laboratory meetings, research meetings, other research studies, and appropriate conferences.
- The candidate has publication plans that would support his career development.

Weaknesses

- The goal to develop expertise in live video intervention delivery does not feel sufficiently ambitious. The adaptation of an intervention to live video is appropriate but very narrow and perhaps not complex enough to justify its own training goal.
- The career plan is narrow and relies heavily on the candidate’s current expertise.
- It is unclear that the visit to the specialty mTBI clinic would be sufficient to develop mastery in the unique issues of mTBI patients. Also, this mentor is remote.
- The candidate did not discuss training, coursework, or mentorship in relevant measurement areas. He would rely heavily on measures currently used in his mentor’s study but given that his population is different other measures may be appropriate. Further, there was little discussion about the role of appropriate measurement for RCTs.

3. Research Plan:
Strengths

- The intervention adaptation strategy is well-thought out involving live video qualitative interviews with 20 subjects on treatment preferences, and an open pilot with five subjects.
- The RCT sample size of the intervention is scientifically justified. It is based on a sound theoretical framework.
- The justification for selecting the target population was explicit and highly significant. The candidate describes the link of mTBI with anxiety and activity avoidance as mediators.
- Good preliminary studies.

Weaknesses

- It’s not clear why the candidate’s goal is a UG3/UH3 grant rather than a R01.
- The candidate critiques the current standard of care for mTBI, that the wait and see and rest until symptom-free approach contributes to symptom persistence and disability. These assertions are not sufficiently scientifically justified and it raises questions about the ability to implement the intervention if it conflicts with basic tenets of the mTBI paradigm.
- There is insufficient attention to the role of smoking and drinking behavior in the model and in measurement plans.
- It is not clear that the intervention would be modified or adapted as a result of the candidate’s activities, especially when looking at the session structure on page 79. The candidate renames some areas of the toolkit or expands on content but the actual changes are not clear.
- It is not clear how measurement instruments would be adapted and piloted. Also, the role of headache pain would not be well-measured. There could be heterogeneity on time since injury, but this would also not be well-measured.

4. Mentor(s), Co-Mentor(s), Consultant(s), Collaborator(s):
Strengths

- The mentors have sufficient background in necessary areas. Well-balanced.

Weaknesses

- No major weaknesses noted.

5. Environment and Institutional Commitment to the Candidate:
Strengths

- The institution is providing strong research facilities to the candidate. He is a member of the Integrated Health Clinical Research Program (IHCRP), a relatively new program at Harvard Medical School, directed by his primary mentor. He would be recruiting in the Concussion Clinic.
- The institution has expertise in the video technology to be used by the candidate.
- The research topic of the candidate is integral to the IHCRP and modeled after existing research at this Institute.
- If the award was made, the candidate would have 90% protected time to carry out his research and training.

Weaknesses

- The commitment of Harvard University and MGH to the candidate is unclear. His job title is post-doctoral fellow and there was no discussion of him moving to a faculty track. The institutional commitment letter said he would be offered a three-year full-time salary as a clinical assistant in Psychology and an Instructor at Harvard medical school, and it stated this appointment is not contingent on the award, however the appointment had not appeared to of occurred at the time of the application.

Study Timeline
Strengths

- The timeline was detailed and reasonable.

Weaknesses

- There was inadequate anticipated and discussed challenges, which was a weakness.

Protections for Human Subjects

- Acceptable Risks and Adequate Protections

Data and Safety Monitoring Plan (Applicable for Clinical Trials Only):

- Acceptable
  - There are three independent Data and Safety Monitoring Board (DSMB) members, but it was not clear from where these members would be recruited.

Inclusion Plans

- Sex/Gender: Distribution justified scientifically
- Race/Ethnicity: Distribution justified scientifically
- For NIH-Defined Phase III trials, Plans for valid design and analysis: Not applicable
- Inclusion/Exclusion Based on Age: Distribution not justified scientifically
- The candidate plans recruit 18-24-year-olds. The clinic he is recruiting from has younger clients. The exclusion of younger clients is not scientifically justified.

Vertebrate Animals
Not Applicable (No Vertebrate Animals)

Biohazards
Not Applicable (No Biohazards)

Resubmission
Not Applicable

Revision
Not Applicable

Training in the Responsible Conduct of Research
Unacceptable
Comments on Format:

- It is not clear there is sufficient face-to-face discussion that would be ongoing.

Comments on Subject Matter:

- The number of modules was described, but not the content.

Comments on Faculty Participation:

- Adequate.

Comments on Duration:

- Not a commitment to last the length of the potential award. Most activities would occur in the first year.

Comments on Frequency:

- There is no commitment to a minimal number of hours each year.

Select Agents
Not Applicable (No Select Agents)

Resource Sharing Plans
Acceptable

Authentication of Key Biological and/or Chemical Resources
Not Applicable (No Relevant Resources)

Budget and Period of Support
Recommend as Requested

CRITIQUE 2

Candidate: 2
Career Development Plan/Career Goals /Plan to Provide Mentoring: 4
Research Plan: 6
Mentor(s), Co-Mentor(s), Consultant(s), Collaborator(s): 3
Environment Commitment to the Candidate: 1

Overall Impact:
This career development application focuses on developing a candidate with an expertise in development and rigorous testing of mind-body interventions, specifically focused on reducing long-term symptoms or problems after injury or illness. The candidate has a strong clinical background in mind-body interventions/approaches. He has demonstrated an interest and aptitude for clinical research related to the development of mind-body interventions. The stated long-term career goal of the candidate is to become an independent investigator with a focus on the development and evaluation of novel, feasible, and scalable mind-body interventions for medical populations, with a particular focus on the prevention of chronic problems. The candidate's stated training goals are to develop skills and expertise in qualitative data collection and analysis, technology-based interventions, mind-body intervention development and adaptation, and the rigorous conduct of mind-body clinical trials. The application's research plan utilizes college-aged individuals with concussion/mild traumatic brain injury (mTBI) and anxiety as a model to evaluate the use of a specific mind-body intervention to prevent chronic symptoms after injury. The candidate has demonstrated the drive and potential to become an independent investigator. He has assembled an overall strong mentoring team and is working in a supportive environment. The research project is synergistic with the candidate's career development plan. Some potentially missing or underdeveloped components of the career development plan and underdeveloped or unclear aspects of the research approach limit some of the overall enthusiasm for this application; thus, the overall impact of this project is considered to be medium.

1. Candidate:
Strengths

- The candidate has a history of prior training in mind-body related interventions, cognitive neuroscience of yoga and meditation, and relationship of mind-body interventions to injury and pain.
- Cultivating interest in mind-body program over many years.
- Two post-doctoral fellowships in related areas.
- Involved in a good number of manuscripts, several as first-author.
- Experience in clinical development of mind-body interventions and associated pilot clinical trials.
- Some neuroscience and imaging experience related to understanding biomarkers associated with mind-body interventions.
- The principal investigator on one pilot grant and experience with participating in other research projects.

Weaknesses

- It is unclear the extent of regulatory and administrative experience in leading research projects, specifically clinical trials. (minor)
- The extent of experience in clinical trial design and analysis is unclear. (minor)

2. Career Development Plan/Career Goals & Objectives:
Strengths

- The application overall identifies appropriate conferences and training activities/courses related to the project that address training goals.
- The career development plan and research project are synergistic.
- The career development plan focuses on self-identified areas of needed development: qualitative research methods, use of technology to delivery interventions, and clinical trial methodology, specifically adaptive designs are mentioned.
- The candidate would be integrated into regular laboratory meetings.
- A plan for ethical training related to research is in place.
- Skills learned would be generalizable to other populations and disorders.

Weaknesses

- The extent of novel/unique clinical trial design and analysis training is unclear. The career development plan was more focused on qualitative and mixed methods. (medium)
- Unclear details regarding the process for monitoring and evaluating the candidate’s research and career development progress, besides noting participation in intermittent meetings. (medium)
- Telehealth delivery seems to be an important goal of the candidate; however, the telehealth training plan seems underdeveloped. (medium)
- The extent of data management training is unclear. (medium)
- The extent of statistical training related to clinical trials is unclear. (medium)

3. Research Plan:
Strengths

- The scientific premise is well-stated regarding the goal of targeting individuals with pre-morbid anxiety.
- Innovative use of a mind-body intervention to prevent chronic symptoms.
- Proposed intervention has been used in other populations successfully.
- A naïve research coordinator would be reviewing scores and data.
- Adequate plan for qualitative coding and review.
- Adequate plan to evaluate intervention fidelity.
- Appropriate structure for intervention adaptation and development.

Weaknesses

- There is concern with the feasibility of recruiting and the retention of 60 individuals in a RCT over a two-year period if barriers are encountered. (minor)
- Unclear frequency of the four Toolkit for Optimal Recovery (TOR) sessions, e.g., once per week, four-consecutive days, etc. (minor)
- Unclear rationale for Health Enhancement as a control condition, specifically when trial design and assessment do not appear to use the control condition in the analyses in a meaningful way besides assessing preliminary “efficacy”. Also, it was unclear whether a Health Enhancement control would even be appropriate for use in a larger efficacy trial or whether a wait-list condition or other design might be more appropriate. (medium)
- Unclear how this pilot study would be used to assist in the selection of optimal outcome measures for future studies. (medium)
- Unclear if other trial designs were considered, e.g., dosing intensity of TOR, feasibility of competing TOR. (medium)
- Age range, time since injury, and, likely mechanism of injury, are restricted based on inclusion/exclusion criteria; thus, limiting generalizability and ability to determine if other characteristics might be important to consider when developing future inclusion/exclusion criteria for a larger efficacy trial. (medium)
- Unclear if the severity of anxiety would be characterized/assessed in regards to influence on outcomes. (medium)
- Unclear how time since injury would be characterized/assess, which is likely an important consideration for population selection for a larger study. (medium)
- Unclear how other injury factors and past medical history would be characterized in regards to influence on outcomes, which is likely an important consideration of population selection for a larger study. (medium)

4. Mentor(s), Co-Mentor(s), Consultant(s), Collaborator(s):
Strengths
- Primary mentor has strong training in mind-body and complementary medicine intervention development and implementation.
- The candidate has active and productive relationship with current mentors.
- The primary mentor has a strong history of successful mentoring.
- Strong mentors in the area of mTBI/concussion.
- Mentors’ letters of support indicate strong commitment to the candidate and project.

Weaknesses
- Appears to be lacking in-depth clinical trial design and statistical analysis mentoring. (medium)

5. Environment and Institutional Commitment to the Candidate:
Strengths
- The institution is supporting the time of investigator appropriately.
- Effort supported for research assistance and others would be supported in-kind by the institution.
- The candidate appears to be well-integrated into the Integrated Brain Health Clinical and Research Program.
- Strong institutional letters of support.

Weaknesses
- No major weaknesses noted.

Study Timeline
Strengths
- Seems well-integrated into the institutional structure and is taking advantage of local resources to optimize potential for success of the project.
- The timeline of project seems feasible but allows for little wiggle room.

Weaknesses
- No major weaknesses noted.

Protections for Human Subjects
- Acceptable Risks and Adequate Protections
  - An acceptable plan is documented.

Data and Safety Monitoring Plan (Applicable for Clinical Trials Only):
- Acceptable
  - An acceptable plan is documented.

Inclusion Plans
- Sex/Gender: Distribution justified scientifically
- Race/Ethnicity: Distribution justified scientifically
- For NIH-Defined Phase III trials, Plans for valid design and analysis: Not applicable
- Inclusion/Exclusion Based on Age: Distribution justified scientifically
- Areas addressed appropriately scientifically.

Vertebrate Animals
Not Applicable (No Vertebrate Animals)

Biohazards
Not Applicable (No Biohazards)

Training in the Responsible Conduct of Research
Acceptable
Comments on Format:
- Would participate in acceptable training formats.

Comments on Subject Matter:
- The proposed subject matter covered seems appropriate.

Comments on Faculty Participation:
- Acceptable, but more details could have been provided on the interactions planned.

Comments on Duration:
- Seems like the minimum hours of instruction would be met.

Comments on Frequency:
- Seems like the minimum frequency would be met.

Budget and Period of Support
Recommend as Requested

CRITIQUE 3

Candidate: 2
Career Development Plan/Career Goals /Plan to Provide Mentoring: 4
Research Plan: 4
Mentor(s), Co-Mentor(s), Consultant(s), Collaborator(s): 3
Environment Commitment to the Candidate: 1

Overall Impact:
This K23 application covers career development for a very strong principal investigator candidate who plans to grow his knowledge of traumatic brain injury (TBI) and mind-body interventions to develop a telehealth-based intervention for mild (m)TBI-related anxiety that may forestall the transition of mTBI

from acute to chronic. This application is from a strong candidate for a K23 award that has already demonstrated strong potential for success. Described mentors are very strong and the plan for career development includes an outstanding curriculum in qualitative methods. The described study is interesting and does fill a meaningful gap in mTBI research. There are a few concerns that diminish enthusiasm for this K23 application. First, some of the most meaningful and in-depth mentoring would come from a mentor with whom the candidate already has experience. Second, the depth and breadth of mTBI mentoring may be insufficient to give the candidate the expertise needed to build a career in this area. Third, the underlying anxiety model of mTBI chronicity is not complete and does cast some doubt on the success of the trial. Fourth, the candidate has not given a compelling reason why the pain-based intervention [the Toolkit for Optimal Recovery (TOR)] fills a meaningful gap that could not already be accounted for by available psychotherapeutic interventions for anxiety.

1. Candidate:
Strengths
- The candidate has a strong academic background for the proposed work including a doctorate in Clinical Psychology, and postdoctoral fellowships in psychiatric neuroscience (studying the neuroscience of yoga and meditation) and integrated brain health (studying mind-body interventions for injury and pain).
- The candidate's work with Drs. Lazar and Vranceanu is a strength and demonstrates exposure to strong research laboratories in mind-body interventions. It is worth noting that Dr. Greenberg has developed a number of first-author publications in these laboratories.
- The principal investigator has developed a strong body of work on mind-body training and mindfulness including the influence of mind-body interventions on brain structure and cognition – this work is relevant to the proposed application and provides a good foundation for growth.
- Mentors to date are experienced researchers with established records of accomplishment of federally funded grants and experience mentoring students and postdoctoral fellows who have been successful with funded research including K-awards.
- Dr. Vranceanu has experience with technology-based interventions (including the use of Skype to reach patients), which is relevant to the proposed study in this K23 application.
- The applicant has strong potential for scientific productivity.
- Letters of recommendation come from strong referees who have ample experience with the applicant. Reference letters speak to diligence, problem-solving, and professionalism that will serve the candidate well throughout his career and portend success.

Weaknesses
- Descriptions of the candidate's experience with mTBI are vague ("I was able to observe firsthand the receptivity and benefit of early mind-body treatment for patients with mTBI.") making it difficult to assess his current understanding of mTBI. mTBI is complex [including a host of persisting post-concussion syndromes (PPCS) and psychiatric comorbidities that alter brain functioning and the clinical phenotype of the mTBI patient]. There is a great deal of ongoing work exploring the transition of mTBI from acute to chronic, but there are no firm answers and significant expertise would be needed to master addressing this question.

2. Career Development Plan/Career Goals & Objectives:
Strengths
- Interaction with mentors would be strong and formative (though some parts of the mentor interaction would simply reproduce experience that the candidate already has).
- Training in qualitative methods through the Harvard School of Public Health, Harvard Catalyst and NVivo is strong and important for the proposed research program as part of this K23 application. These skills are likely to benefit the candidate in the future and combining strong curriculum with a real-world test of these skills in the candidate's research study is a strength.

Weaknesses

- Training for mTBI is vague and unconvincing. TBI is a complex clinical phenomenon and training in mTBI is arguably the most important part of this K23 application because this is the candidate's greatest weakness and the focus of his research plan. The candidate reports weekly to monthly meetings with Dr. Iverson, who is a strong mentor, but gives little detail about how time with Dr. Iverson would be structured and used to help him establish enough expertise in mTBI to complete his research and direct his career. More frequent mTBI consultation throughout would strengthen the plan.
- The candidate does not have a formal training goal attached to mentorship from Dr. Silverberg, who is a strong subject matter expert in mTBI at the University of British Columbia. He is already meeting with Dr. Iverson, so it is hard to tell how infrequent meetings with Dr. Silverberg would meaningfully add to his career development plan. The candidate would have monthly hour-long Zoom meetings with Dr. Silverberg.
- The candidate would attend weekly research meetings with Dr. Vranceanu's Integrated Brain Health Clinical and Research Program (IBHCRP) laboratory as part of his career development plan. He is already attending these meetings, so it is likely that continuing to do so would not significantly add to his knowledge or skills.
- It is unclear how the candidate would supplement curriculum-based training on qualitative methods with personal mentoring. Active qualitative research can be challenging and supplementing his strong curricular training with prospective mentoring in qualitative methods would have strengthened the career development plan. This concern is diminished quite a bit by the candidate's experience with Dr. Vranceanu, who would assist in the qualitative components of the research program and has experience in qualitative research.

3. Research Plan:
Strengths
- Using a mind-body intervention to address TBI symptom persistence is valuable and innovative.
- Establishing an efficacious mind-body intervention for mTBI that is delivered by live video is a significant innovation that would benefit a patient population that may be disabled and unable to attend clinic-based care appointments. The proposed TOR-C program would be accessible (though there is concern about potential PPCS symptom exacerbation with prolonged viewing of a computer screen; i.e., headache exacerbation).
- Preliminary data showing anxiety as an indirect predictor of PPCS is interesting and somewhat supports the proposed model of anxiety in mTBI. However, it is unclear how many other potential variables were accounted for in mTBI symptom progression so unique variance attributable to anxiety is difficult to estimate from the data provided.
- The plans for qualitative methods and refinement are solid. The collaborators have experience with the Vidyo software to be used so feasibility of Vidyo interviews is not a significant concern.

Weaknesses
- The research plan would investigate 4-session live video TOR to treat anxiety in individuals with mTBI. The ultimate goal of this research is to prevent PPCS in college students with mTBI. The proposed model of anxiety and mTBI symptoms makes sense. However, mTBI is very complex and the candidate's proposition that improvement in anxiety can prevent PPCS is overstated without additional preliminary data.
- Because anxiety is the clinical target of TOR-C, more could have been done to bolster the need for TOR-C as an anxiety intervention in mTBI. Pharmacological interventions (especially benzodiazepines) are problematic and risky in mTBI, so the proposed mind-body intervention is attractive because of its low risk. However, there are already a number of efficacious, manualized, non-pharmacological interventions for anxiety that could have been used in this study instead of a program adapted from pain management. A stronger case needed to be made to specifically support TOR-C over these other proven programs.

4. Mentor(s), Co-Mentor(s), Consultant(s), Collaborator(s):

Strengths
- The candidate would receive unparalleled mentorship in mind-body interventions (from Drs. Vranceanu and Yeh).
- The candidate would meet with two strong TBI experts (Drs. Iverson and Silverberg).

Weaknesses
- Mind-body mentorship would be driven by Dr. Vranceanu, a mentor with whom the candidate already has quite a bit of experience in his postdoctoral fellowship. This component of mentorship may not lead to novel skills or content knowledge development.
- The candidate would receive biostatistical consultation from Dr. Macklin but does not appear to have any planned mentoring in data analytic planning and interpretation from his meetings with Dr. Macklin; this seems like a missed opportunity.

5. Environment and Institutional Commitment to the Candidate:
Strengths
- Facilities and resources through Harvard University and MGH are excellent and the candidate plans to make effective use of these resources for his career development.

Weaknesses
- No major weaknesses noted.

Study Timeline
Strengths
- The timeline looks reasonable for the stated work.

Weaknesses
- Only nine months from open pilot to analyze and complete the institutional review board process for the pilot RCT seems a little tight.

Protections for Human Subjects
- Acceptable Risks and Adequate Protections
    - The plan is well-developed. No concerns noted.

Data and Safety Monitoring Plan (Applicable for Clinical Trials Only):
- Acceptable
    - Minimal risk.

Inclusion Plans
- Sex/Gender: Distribution justified scientifically
- Race/Ethnicity: Distribution justified scientifically
- For NIH-Defined Phase III trials, Plans for valid design and analysis: Not applicable
- Inclusion/Exclusion Based on Age: Distribution justified scientifically
- Even split on sex; sample is mostly white, resembling the MGH population; only 18-24 age range would be admitted - patients outside this age range would benefit from the intervention under study.

Vertebrate Animals
Not Applicable (No Vertebrate Animals)

Biohazards
Not Applicable (No Biohazards)

Training in the Responsible Conduct of Research
Acceptable
Comments on Format:
- In-person, weekly meetings.

Comments on Subject Matter:
- Subject matter covered in training is appropriate.

Comments on Faculty Participation:
- Faculty are very involved. A strength of this application.

Comments on Duration:
- Duration seems appropriate.

Comments on Frequency:
- For the most part, the candidate is meeting with faculty at regular frequency.

Resource Sharing Plans
Acceptable
- Media outreach and the MGH Development Center; the candidate has a history of success with dissemination through public media.

Budget and Period of Support
Recommend as Requested
- The budget appears reasonable for the described work.

CRITIQUE 4

Candidate: 3
Career Development Plan/Career Goals /Plan to Provide Mentoring: 4
Research Plan: 5
Mentor(s), Co-Mentor(s), Consultant(s), Collaborator(s): 3
Environment Commitment to the Candidate: 1

Overall Impact:
This application addresses significant needs to identify the predictors and treatment modalities for concussed patients at risk for developing chronic post-concussive symptoms or syndrome (PCS). Concussed individuals with concurrent anxiety in acute phase of injury was identified as a major “target” for the proposed studies. This is overall a good project from a qualified team of investigators and promising candidate. However, several serious issues with the career development plan, lack of conceptual clarity, and research methodology concerns reduced enthusiasm for this application.

1. Candidate:
Strengths
- The candidate has sufficient experience in clinical psychology, adequate academic background, clinical training and current clinical experience - all these have the potential to develop the candidate as an independent and productive clinical researcher.
- Overall strong publication records (17 publications, 3 submitted or under review), oral conference presentations (n=9), etc., on using mind-body interaction approaches to prevent transition from acute to chronic clinical symptoms and disability.

Weaknesses
- There seems to be a lack of commitment and passion to explore the unique nature of concussive injuries, its phenotypes, mechanisms, underlying neural substrates, neurological sign and symptoms, and classification of injury.
- The lack of familiarity with multi-factorial and multi-modal presentation of concussive injury prognosticating evolution of injury and predicting the risk for developing post-concussive syndrome (PCS).

- Lack of candidate's research and publication record in the area of concussive injury. Virtually no research publications on concussion in refereed journals, which is a serious limiting factor questioning the candidate's commitment to be an independent researcher in the field of mTBI.
- Overall, it is questionable the candidate's capacity to organize, manage, and implement the proposed programs and studies in the mTBI field.

2. Career Development Plan/Career Goals & Objectives:
Strengths

- Career goals, such as adopting the live video mind-body program in the field of concussive injury and acquire training in methods of qualitative analysis of interviews, may have potential to contribute to the scientific development of the candidate and may potentially lead to scientific independence in the field of clinical/telehealth psychology.

Weaknesses

- There is no indication and/or intention in the career development plan to attend workshops, seminars, and/or conferences to acquire and disseminate knowledge in the TBI field.
- There is no plan for coursework with a specific focus on mTBI, at odds considering the scope and content of this application.
- There don't appear to be plans or goals to understand neurological aspects of concussive injury. It should be noted that concussion is the "most puzzling *neurological* disorder facing sports medicine today…"
- The candidate has published a (first-author) a manuscript on the "qualitative analysis of focus groups and exit interviews of patients with chronic pain". Thus, there is lack of clarity in the career development plan as to why additional training would be needed to achieve training goal number two.

3. Research Plan:
Strengths

- There is sufficient evidence of implementing live video mind-body interaction procedures for patients with orthopedic injuries with chronic pain.
- Attempts and some preliminary studies from the research team to assess the feasibility of the proposed treatment protocol for mTBI patients.

Weaknesses

- There is a lack of conceptual clarity in the Research Plan regarding the feasibility of a live video mind-body program in chronic pain for preventing the development of PCS after acute TBI.
- There appears to be a lack of consideration in the Research Plan that other than TBI symptoms other than anxiety may lead to the development of chronic PCS. The claim that "…anxiety has emerged as one of *the most robust predictors of progression to persistent symptoms after mTBI"* is not well-supported by the broad mTBI literature. Of course, anxiety is an important contributor to concussive symptoms presentation, but, clinical and conceptual significance of this application solely based on concussion/anxiety linkage as the most "robust predictor" of PCS needs further justification.
- There is no clear mechanistic hypothesis elaborated on how mild-body intervention with specific focus on anxiety may be used as a preventive strategy of TBI-persistent symptoms beyond anxiety per se. Again, multi-modal neurocognitive, behavioral, and emotional signs and symptoms that may be present in the chronic phase of TBI are not considered.
- The achievement of Specific Aims 2 and 3 depends on the outcome of Specific Aim 1. There doesn't appear to be a back-up plan in the research protocol if "treatment needs" aren't identified.
- If the proposed live video mind-body interaction techniques are "well received by patients, cost-effective, and has comparable efficacy to face-to-face interventions" it is not clear the novelty and need for addressing Aims 1-3.

- The majority of concussive injury clinical signs and symptoms are transient and resolved within acute (typical recovery) and sub-acute phases of injury. “Atypical” cases (prolong symptoms far beyond the acute phase of injury) are rare and may have differential etiology, history, etc. There are no details in the Research Plan to explore and consider differential outcomes from concussive injury (e.g., complicated versus non-complicated).
- There is growing public concern regarding the consequences of repetitive concussive and sub-concussive head acceleration events (HAEs). These may be a significant factor predicting transition from acute to chronic phases of injury. The lack of consideration of this factor may confound the feasibility of adopting live video mind-body approach to mTBI.
- As written, “Mind-body interventions effectively treat individual symptoms common to mTBI (e.g., insomnia, headache, and fatigue) and anxiety. They have also been shown to reduce stigma associated with traditional mental health referrals and are already popular among patients with neurological concerns.” Thus, it is not clear the novelty of the proposed studies within the scope of Aims 1-3.
- The vast majority of concussed collegiate athletes experiences light sensitivity and exaggerated other symptoms (e.g., headache, etc.) while looking at the computer or watching the television screen in the acute phase of injury. It is not clear how the proposed live video mind-body intervention might interfere or compromise the standard clinical management of mTBI.
- There doesn’t appear to be any preliminary pilot studies conducted by the candidate directly related to the feasibility of Specific Aims 1-3.
- There is a lack of demographic information about human subjects inclusion, especially mechanisms of injury, history of previous concussions, time since concussive injury, its severity, etc., in the proposed study design.

4. Mentor(s), Co-Mentor(s), Consultant(s), Collaborator(s):
Strengths
- Excellent primary mentor and highly qualified collaborators in the area of clinical psychology, telehealth, neuropsychology of mTBI, and methods of qualitative analysis fully capable of fostering of the candidate’s development as an independent investigator.

Weaknesses.
- A lack of mentor(s) with specific expertise in clinical neurology is a weakness, which is a serious limitation considering the focus of this application on mTBI.

5. Environment and Institutional Commitment to the Candidate:
Strengths
- Strong environment and institutional commitment to the candidate.

Weaknesses
- No major weaknesses noted.

Study Timeline
Strengths
- Clearly elaborated. There were no concerns.

Weaknesses
- No major weaknesses noted.

Protections for Human Subjects
- Acceptable Risks and Adequate Protections

Data and Safety Monitoring Plan (Applicable for Clinical Trials Only):
- Acceptable

Inclusion Plans
- Sex/Gender: Distribution justified scientifically

- Race/Ethnicity: Distribution justified scientifically
- For NIH-Defined Phase III trials, Plans for valid design and analysis: Not applicable
- Inclusion/Exclusion Based on Age: Distribution justified scientifically

Vertebrate Animals
Not Applicable (No Vertebrate Animals)

Biohazards
Not Applicable (No Biohazards)

Training in the Responsible Conduct of Research
Acceptable
Comments on Format:
- Acceptable format.

Comments on Subject Matter:
- Acceptable subject matter.

Comments on Faculty Participation:
- Acceptable faculty participation.

Comments on Duration:
- Acceptable duration.

Comments on Frequency:
- Acceptable frequency.

Resource Sharing Plans
Acceptable

Budget and Period of Support
Recommend as Requested

THE FOLLOWING SECTIONS WERE PREPARED BY THE SCIENTIFIC REVIEW OFFICER TO SUMMARIZE THE REVIEWERS' WRITTEN CRITIQUES ON THE FOLLOWING ISSUES:

PROTECTION OF HUMAN SUBJECTS: ACCEPTABLE

INCLUSION OF WOMEN PLAN: ACCEPTABLE

INCLUSION OF MINORITIES PLAN: ACCEPTABLE

INCLUSION ACROSS THE LIFESPAN PLAN: UNACCEPTABLE. The candidate plans to recruit individuals in the 18-24-year-old age range. The exclusion of younger individuals was not scientifically justified.

COMMITTEE BUDGET RECOMMENDATIONS: The budget was recommended as requested.

Footnotes for 1 K23 AT010653-01; PI Name: Greenberg, Jonathan

NIH has modified its policy regarding the receipt of resubmissions (amended applications). See Guide Notice NOT-OD-14-074 at http://grants.nih.gov/grants/guide/notice-files/NOT-OD-

14-074.html. The impact/priority score is calculated after discussion of an application by averaging the overall scores (1-9) given by all voting reviewers on the committee and multiplying by 10. The criterion scores are submitted prior to the meeting by the individual reviewers assigned to an application, and are not discussed specifically at the review meeting or calculated into the overall impact score. Some applications also receive a percentile ranking. For details on the review process, see http://grants.nih.gov/grants/peer_review_process.htm#scoring.

MEETING ROSTER

The roster for this review meeting is displayed as an aggregated roster that includes reviewers from multiple AT Special Emphasis Panels Meetings
for the 2019/10 council round.
This roster for AT is available at:
http://public.era.nih.gov/pubroster/Reports?DOCTYPE=SEP&DESFORMAT=PDF&AGENDA_SEQ_NUM_P=372170
